# An optimization model for monthly time-step drilling schedule under planned field production

**DOI:** 10.1016/j.heliyon.2024.e28979

**Published:** 2024-04-09

**Authors:** Jingyun Ouyang, Shaoyang Geng, Shuo Zhai

**Affiliations:** a Chengdu North Petroleum Exploration and Development Technology Co., Ltd., Chengdu, Sichuan, China; b College of Energy, Chengdu University of Technology, Chengdu, Sichuan, China

**Keywords:** Drilling schedule optimization, Production optimization model, Gradient descent method, Development plan optimization

## Abstract

The field production profile over the yearly horizon is planned for a balance between economy, security, and sustainability of energy. An optimal drilling schedule is required to achieve the planned production profile with minimized drilling frequency and summation. In this study, we treat each possible production process of each well as a dependent time series and the basic unit. Then we ensemble all of them into a tensor. Based on formulated tensor calculation and Lasso regularization, a linear mathematical optimization model for well drilling schedule was developed. The model is aimed at minimizing production profile error while optimizing drilling frequency and summation. Although the model proposed in this work requires more memory consumption to be solved using a computer, it is assured as a linear model and could be numerically globally solved in a stable and efficient way using gradient descent, avoiding complex nonlinear programming problems. Main input data and parameters involved in the model are analyzed in detail to understand the effects of different production parameters on the drilling schedule and production profile. The proposed model in this work can evaluate the manual drilling schedule and automatically generate an optimized drilling schedule for the gas field, significantly reducing development plan formulation time.

## 1 Introduction

As the population and the economy grow, demand for petroleum production is increasing [1]. China has repeatedly stressed the need to increase exploration and development efforts to enhance oil and natural gas reserves and production, ensuring national energy security and meeting residential energy demand [2,3]. The exploration and development of oil and gas resources should consider both energy security and economic demand [4]. However, for China, which lacks oil and gas resources, planned drilling to meet the country's demand for oil and gas resources takes precedence over achieving economic benefits. One of the pivotal steps of the field plan is to finalize the drilling schedule [5], determining the timing and the relevant number of drillings—specifically, how many wells should be drilled in a given month [6].

Scientific and rational drilling timing poses a complex challenge. Calculating the drilling number for the next year based solely on the target production is not accurate [7,8]. Comprehensive consideration of construction, stability, and the decline of field production is necessary. Assuming the target field production is achieved, optimal drilling timing is expected to optimize the frequency and summation of drilling [9]. Research on the relationship between economics and drilling has advanced significantly. Earlier studies have explored methods to optimize drilling scheduling concerning placement and timing [10]. In fact [11], highlighted that various combinatorial problems, particularly those involving scheduling and allocation, could be formulated as linear integer programming problems. However, when it comes to drilling scheduling, the situation becomes intricate [12]. proposed a continuous-time mathematical formulation for batch and continuous processes applicable to describe oil and gas field production processes [13]. explored flexible processing systems and proposed a new continuous-time model for long-term planning in offshore gas field development [14]. presented a two-level continuous-time modeling and optimization approach. This approach introduced the concept of event points, allowing well platforms to come online at potentially any time within the continuous horizon under consideration [15]. presented a workflow for determining the optimal type and location of new wells using an optimization implementation [16]. developed a mixed integer nonlinear programming model for offshore oilfield development, encompassing floating production, storage, well drilling, and production rates.

Previous work reduces the risk of geological uncertainty and increases the return on the drilling program [17,18]. However, their optimal solution is to maximize the value of the remaining reservoir in the shortest possible time, rather than focusing on the proper sequence of drilling to ensure the target production [19,20] developed a model based on a genetic algorithm and a reservoir simulator for optimal drilling scheduling [21]. investigated modeling sequence-dependent switchovers for uniform discrete-time scheduling problems. Their formulation used memory operation logic variables to track the temporal unit-operation events within the scheduling horizon for each unit [22]. presented a dynamic, holistic, and integrated approach that involved decisions on the order, placement, timing, capacities, and allocations of new well drillings and surface facilities [23]. used a sequential decision model to obtain the optimal drilling strategy and applied the analysis of this strategy to determine the optimal number of wells and corresponding locations [24]. compared productivity, associated costs, and economic revenues to evaluate optimal drilling and completion conditions. Concentrated and dense drillings in a short period may result in excess production capacity, potentially exceeding the transport capacity of field surface facilities and decreasing the net present value due to higher cash costs in the early period [25]. Insufficient drilling will result in a shortage of petroleum production. Thus, an optimal drilling timing schedule is needed to balance the security of petroleum supply and economic value [26,27].

In this study, the goal is to satisfy the planned field production profile as closely as possible with the minimum drilling frequency and total number of drillings. Therefore, we propose an optimization model to optimize the production summation of individual wells, aiming to meet the planned production. The model aims to minimize the production profile error while optimizing the frequency and total number of drilled wells.

## 2 Methodology

### 2.1 Problem statement

**Fig. 1:**
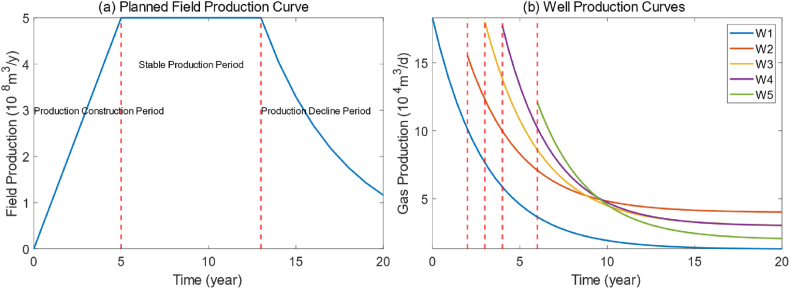
Gas production of different wells and planned field production.

During field development planning, the target field production profile—a time series of annual production—is determined by the geological and economic settings, transmission capacity of surface facilities, and planned field production. In planned field production, the life cycle of a field is typically divided into three stages: production construction period, stable production period, and production decline period, as shown in Fig. 1(a). To achieve the planned oilfield production, the number of wells required each year varies based on the different initial production and decline rate of gas wells, as shown in Fig. 1(b). The number of wells drilled per month must be determined based on the production profile of each well. Excessive drilling in a certain year may lead to overflow beyond the planned production for the year and could surpass the capacity of facilities. Conversely, an inadequate number of wells may result in a failure to reach the target production for the year. Additionally, frequent and concentrated well drilling may lead to an unexpectedly rapid production decline of the field compared to the target profile.

We assume the production profile of the field for the next 20 years, as shown in Fig. 1(a). Due to the actual geology of the field, approximately 100 wells are expected to be drilled within the study area over 7 years. There would be (7 × 12)^100^ possibilities to be adopted for monthly drilling schedule, let alone more consideration about optimized drilling frequency and summation.

**Fig. 2:**
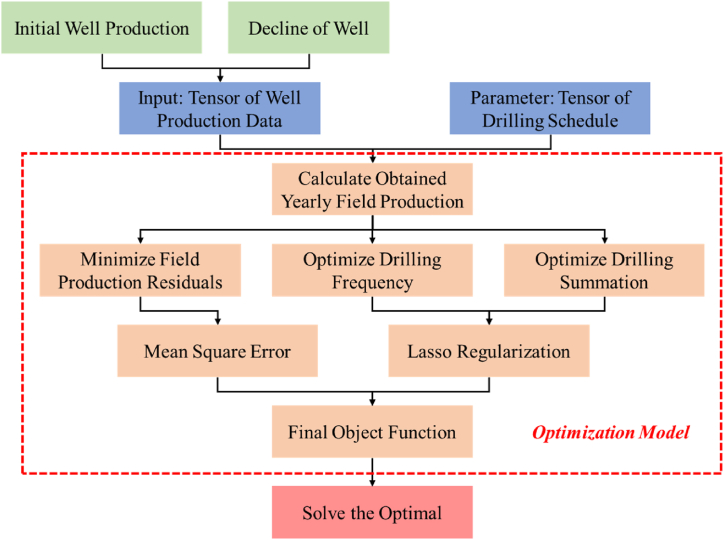
Workflow of drilling schedule optimization.

In the past, during the preparation and implementation of the gas field development plan, the drilling schedule heavily relied on experience of engineers, demanding considerable time and effort. Therefore, our objective is to establish a monthly optimization model for the drilling schedule based on the gas well production profile and the planned gas field production. Here, we consider the production profile of a single gas well as a dependent time series and the basic unit. Subsequently, we aggregate them into a tensor. To minimize the error between calculated field production and planned field production, we employ tensor computation and Lasso regularization to optimize the drilling frequency and total number of wells. The optimization workflow is illustrated in Fig. 2.

### 2.2 Field production profile

Let the drilling years limit ($Y_{dl}$) and production year limit ($Y_{pl}$) is $Y_{dl}$ and $Y_{pl}$. And their monthly counterparts are $T_{dl}=12Y_{dl}$ and $T_{pl}=12Y_{pl}$, respectively.

Given the number of potential drilled well *N*, the predicted future production profiles of the *N* wells can be denoted as a tensor **M** (Eq. (1)):

(1) $$M=\left\{M_{N_{i},j}\right\}$$

where i=1,2,…,N and $j=1,2,\ldots ,T_{pl}$; $M_{N_{i},j}$ is the *j* month production of *i* well.

The production profile of the field planned is given by a tensor **P** (Eq. (2))：

(2) $$P=\left\{P_{y}\right\}$$

where $y=1,2,\ldots ,Y_{pl}$; $P_{y}$ is the annual production target of *y* year.

Assume that *i* well will be drilled on *k* month in the future, each of its monthly productions from 1 to k−1 month will be 0, and those from *k* to $T_{pl}$ month will be $M_{i,1}$ to $M_{i,T_{pl}-k+1}$. All production possible profiles of the *N* wells with drilled-month considered is given by a 3-dimention tensor **G** with length of $\left(N\text{,}T_{pl},T_{pl}\right)$, as shown in Eq. (3):

(3) $$G=\left\{G_{i,k,m}\right\}=\left\{ \begin{array}{c} 0,m<k\\ G_{i,k,m},m\geq k \end{array}\right.$$

where i=1,2,…,N; $k=1,2,\ldots ,T_{dl}$; $m=1,2,\ldots ,T_{pl}$; $G_{i,k,m}$ is the predicted *m* month production of *i* well with the assumption that it will be drilled on *k* month.

The drilling timing schedule is given by a 3-dimention tensor **D** with length of $\left(N\text{,}T_{dl},1\right)$ in Eq. (4):

(4) $$D=\left\{D_{i,k,1}\right\}$$

where $i=1,2,\ldots ,N;\;k=1,2,\ldots ,T_{dl}$; $D_{i,k,1}=1$ means the *i* well will be drilled on *k* month; $D_{i,k,1}=0$ means the well will not be drilled on *k* month. $0<D_{i,k,1}<1$ means a value exception and would be handled in the following.

So, according to drilling schedule tensor D, m month production of the i well is as following:

(5) $$G_{i,m}=Squeeze\left(\sum_{k=1}^{T_{dl}}\left(G_{i,k,m}D_{i,k,1}\right)\right)$$

where Squeeze(∙) is a function that remove axes of length one.

Sum Eq. (5) of all wells, and m month production of the N wells is obtained:

(6) $$G_{m}=\sum_{i=1}^{N}G_{i,m}$$

Annual production of y year is acquired by summing every 12 months on Eq. (6), then we get Eq. (7):

(7) $$Q_{y}=\sum_{m=12y-11}^{12y}G_{m}$$

One of the targets of the optimized drilling schedule is to minimize the residuals between the target and practical field production profiles. A well-defined empirical object function is the Mean Square Error (MSE). Thus, one of the object functions is:

(8) $$L\left(D\right)=\frac{\sum_{y=1}^{Y_{pl}}{\left(P_{y}-Q_{y}\right)}^{2}}{Y_{pl}}$$

With Eqs. (5), (6), (7), Eq. (8) is denoted in means of scalar by:

(9) $$L\left(D\right)=\frac{\sum_{y=1}^{Y_{pl}}{\left(P_{y}-\sum_{m=12y-11}^{12y}\sum_{i=1}^{N}Squeeze\left(\sum_{k=1}^{T_{dl}}\left(G_{i,k,m}D_{i,k,1}\right)\right)\right)}^{2}}{Y_{pl}}$$

The objective of this optimization model is to minimize the error between the planned field production and the calculated results, which means to minimize Eq. (9).

### 2.3 Drilling frequency and summation

We aim to avoid concentrating the drilling schedule in a specific period of time. The drillings are distributed across different times as much as possible. Therefore, we optimize the drilling frequency and total number as follows.

Sum along the first axis, drilling number for each well during $T_{dl}$ month is obtained (Eq. (10)):

(10) $$W=\left\{W_{k}\right\}=\sum_{i}^{N}D_{i,k,1},k=1,2,\ldots ,T_{dl}$$

The other target is to minimize the total number summation and frequency of drilling. The two requirements are equivalent to minimization of summation of tensor **W** and the minimization of each element in **W** (0 is best, if possible), respectively. While the former requires global optimization and the latter resemble greedy method, the combined optimization problem could be complicated. This work proposed a graceful method to implement the two as far as possible.

The constraint penalty function can incorporate the constraint conditions into the objective function so that both the objective function and the constraint conditions can be considered in the optimization problem [28]. The size of constraint penalty factor determines the importance of optimization algorithm in balancing objective function and constraint conditions. In order to make the optimization result meet the planned production, we apply Lasso regularization on parameter tensor $D_{N\times T_{dl}\times 1}$ for summation and frequency minimization, which is supposed to improve the sparsity of $D_{N\times T_{dl}\times 1}$. Lasso Regularization is given by Ref. [29]:

(11) $${\left\|D\right\|}_{1}=\sum_{N_{i}}^{N}\sum_{k}^{T_{pl}}\left|D_{N_{i},k,1}\right|$$

On basis of Eq. (8), with introduction of normalization item Eq. (11), the final object function is given by Eq. (12)：

$$J\left(D\right)=L\left(D\right)+\alpha {\left\|D\right\|}_{1}$$

where α is regularization strength, no unit.

Let $D^{*}$ is the optimal solution of L(D), as shown in Appendix A, the optimal solution of the final object function marked as $\tilde{D}=\left\{{\tilde{D}}_{i,k,1}\right\}$ will be:

(13) $${\tilde{D}}_{i,k,1}=\left\{ \begin{array}{c} 0,\left|D_{i,k,1}^{*}\right|\leq \frac{\alpha }{H}\\ sign\left(D_{i,k,1}^{*}\right)\left(\left|D_{i,k,1}^{*}\right|-\frac{\alpha }{H}\right),\left|D_{i,k,1}^{*}\right|>\frac{\alpha }{H} \end{array}\right.$$

According to Eq. (13), on the basis of the optimal solution of the original object function (i.e., $D_{i,k,1}^{*}$), each element ${\tilde{D}}_{i,k,1}$ of the final optimal solution is adjusted. ${\tilde{D}}_{i,k,1}$_. And if_ $\left|D_{i,k,1}^{*}\right|>\frac{\alpha }{H}$_, a decrement that makes_ ${\tilde{D}}_{i,k,1}$_closer to 0 than_ $D_{i,k,1}^{*}$_would be imposed._

Since more elements will be set to 0 and closer to 0 than the original object function (mean square error of target and practical production profiles), elements in $D^{*}$ will tend to be 0. Thus, elements in **W** will approach 0, which is called sparse in terms of matrix. In summary, the frequency and summation of drilling can be optimized using an extra linear object function (i.e., Lasso regularization).

### 2.4 Optimization model

Consider Eqs. (9), (11) and combine the object functions of production profile error and drilling frequency and number, and the optimization model for drilling timing schedule is given by:

(14) $$\tilde{D}=\left\{{\tilde{D}}_{N_{i},k,1}\right\}=\mathrm{argmin}\left(\begin{array}{c} \frac{\sum_{y=1}^{Y_{pl}}{\left(P_{y}-\sum_{m=12y-11}^{12y}\sum_{i=1}^{N}Squeeze\left(\sum_{k=1}^{T_{dl}}\left(G_{i,k,m}D_{i,k,1}\right)\right)\right)}^{2}}{Y_{pl}}\\ +\alpha \sum_{N_{i}}^{N}\sum_{k}^{T_{pl}}\left|D_{N_{i},k,1}\right| \end{array}\right)$$

### 2.5 Solution workflow to the optimization model

**Fig. 3:**
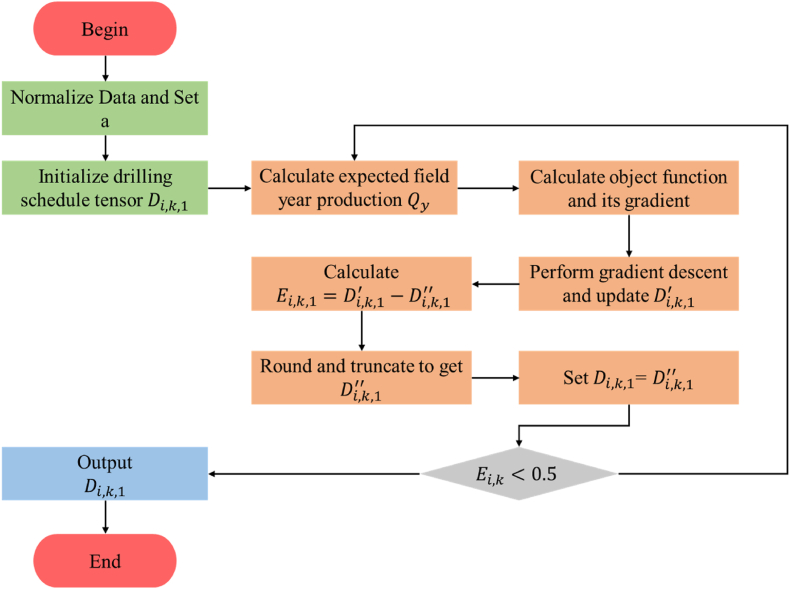
Numerically solving workflow Diagram.

The optimization model developed in this work involves only linear calculations, making it differentiable step by step and solvable using the gradient descent algorithm. However, considering that elements in the optimized drill plan must be either 1 or 0, the solving process of the proposed mathematical model requires improvements in the convergence condition (i.e., stopping criterion) and parameter normalization. This is in addition to the traditional gradient descent algorithm, as shown in Fig. 3.

Details of the workflow are as follows.

Setp (1) . Normalize data and set regularization strength.

As shown in Eq. (12) and Eq. (14), the final object function is multi-target, where L(D) and $\alpha {\left\|D\right\|}_{1}$ are pertinent to different units and magnitudes. Without data normalization, the result of object function would be extremely unstable depending on the unit.

Let,

(15) $$P_{\max}=\max\left(\left\{P_{y}\right\}\right),y=1,2,\ldots ,Y_{pl}$$

Normalize the drilled well monthly production tensor (Eq. (16)):

(16) $$G^{\#}=\left\{G_{i,k,m}^{\#}\right\}=\left\{\frac{G_{i,k,m}}{P_{\max}}\right\}$$

where $i=1,2,\ldots ,N;\;k=1,2,\ldots ,T_{dl};\;i=1,2,\ldots ,T_{pl}$.

We suggest an automatically set regularization strength, that is (Eq. (17)):

(17) $$\alpha =\frac{0.1}{10^{\text{floor}}\left(\log_{10}\;N\right)}$$

where floor(∙) is the function to compute the floor, literally.

This strength of Lasso regularization would ensure that $\alpha {\left\|D\right\|}_{1}$ is of the suitable magnitude. However, the regularization could also be manually set to any rational value even 0, depending on cases.

Setp (2) Initialize the drilling schedule tensor.

Assign all the elements in drilling schedule tensor **D** to 1, which initial value is shown in Eq. (18):

(18) $$D_{i,k,1}=1,i=1,2,\ldots ,N;k=1,2,\ldots ,T_{dl}$$

Setp (3) Calculate the final object function and its gradient.

The object function is calculated by Eq. (19)：

(19) $$J\left(D\right)=\frac{\sum_{y=1}^{Y_{pl}}{\left(P_{y}-\sum_{m=12y-11}^{12y}\sum_{i=1}^{N}Squeeze\left(\sum_{k=1}^{T_{dl}}\left(G_{i,k,m}D_{i,k,1}\right)\right)\right)}^{2}}{Y_{pl}}+\alpha \sum_{N_{i}}^{N}\sum_{k}^{T_{pl}}\left|D_{i,k,1}\right|$$

Take and calculate the derivative of Eq. (19) with respect to each element in tensor D, which is:

(20) $$\frac{\partial J\left(D\right)}{\partial D_{i,k,1}}=\frac{2}{Y_{pl}}\sum_{y=1}^{Y_{pl}}\sum_{m=12y-11}^{12y}Squeeze\left(\sum_{k=1}^{T_{dl}}\left(G_{i,k,m}^{\#}D_{i,k,1}\right)\right)+\alpha \text{sign}\left(D_{i,k,1}\right)$$

Setp (4) . Update drilling schedule tensor using gradient descent algorithm.

Based on the gradient given by calculating Eq. (20), each element in the drilling schedule tensor is updated using gradient descent algorithm [30], which is shown in Eq. (21):

(21) $$D_{i,k,1}^{\prime }=D_{i,k,1}-\eta \frac{\partial J\left(D\right)}{\partial D_{i,k,1}}$$

where η is the learning rate, i.e., the speed of updating parameter. η=0.01 is used in this paper.

Setp (5) Calculate the changed value of each element in drilling tensor between that before and after the gradient descent (Eq. (22)).

(22) $$E_{i,k,1}=\left|D_{i,k,1}-D_{i,k,1}^{\prime }\right|$$

Setp (6) Clip and round element in the drilling tensor.

Because the value of gradients is float and may be extremely enormous, $D_{i,k,1}^{\prime }$ may become float or negative or excel 1 after gradient descent. In fact, a potential well can be only drilled once in the future. So further processing should be applied to $D_{i,k,1}$.

Sum $D_{i,k,1}^{\prime }$ along the drill month axis (Eq. (23)):

(23) $$D_{i,1}^{\prime }=\sum_{k=1}^{T_{dl}}D_{i,k,1}^{\prime }$$

where $D_{i,1}^{\prime }$ is total drilled number of the i well, which is supposed to be 1 or 0, but very probably not during the practical optimization due to the involvement of float and negative value. So, $D_{i,k,1}^{\prime }$ is rectified and turned into $D_{i,k,1}^{\prime \prime }$ according to:

(24) $$D_{i,k,1}^{\prime \prime }=\left\{ \begin{array}{c} \text{clamp}\left(\text{round}\left(\frac{D_{i,k,1}^{\prime }}{D_{i,1}^{\prime }}\right)\right),D_{i,1}^{\prime }\geq 1\\ \text{clamp}\left(\text{round}\left(D_{i,k,1}^{\prime }\right)\right),D_{i,1}^{\prime }<1 \end{array}\right.$$

where round(∙) is integral rounding function. clamp(∙) is truncation function, which turns the negative argument into 0.

With Eq. (24), Only one or less than one element in $D_{i,k,1}^{\prime \prime }$ will be 1.

Setp (7) Check convergence.

For i=1,2,…,N and $k=1,2,\ldots ,T_{dl}$, check every element:

(25) $$E_{i,k,1}<0.5$$

Notice rounding function is applied in step (6), so when Eq. (25) is true,

(26) $$D_{i,k,1}-D_{i,k,1}^{\prime \prime }=0$$

Eq. (26) means that there are not any more improvement in the iteration, where the parameter tensor is convergent.

Setp (8) Reassign the drilling schedule tensor variable (Eq. (27)).

(27) $$D_{i,k,1}=D_{i,k,1}^{\prime \prime }$$

Setp (9) Iterate until convergent.

Repeat step (3)(4)(5)(6)(7)(8), until the stopping criterion in step (7) is reached.

## 3 Results and discussion

**Fig. 4:**
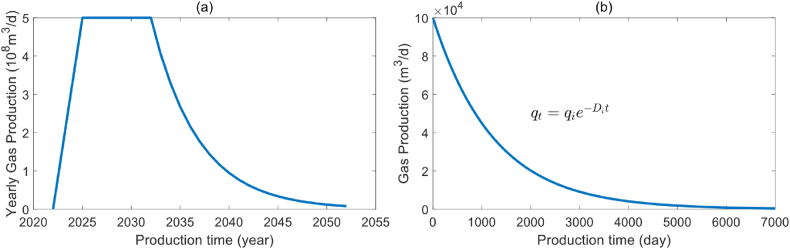
Single well production profile (a) and field production profile (b).

We employ a basic gas reservoir model to analyze the main factors influencing the optimization results. The basic parameters of the gas reservoir model are as follows: As shown is Fig. 4(a), the gas reservoir will commence production in 2023, with the construction period lasting for the first three years, followed by eight years of stable production, nineteen years of decline. The entire life cycle of the gas reservoir will be 30 years. During the stable production period, the annual production target is 5 × 108m3/y, and the limited drilling year is 10 years. Assuming the default parameters of the gas well are as follows: the initial gas production is 10 × 104m3/d, the production decline rate is 0.0005 1/day, and the decline type of production conforms to the exponential decline. The production of each individual well and the planned field production are shown in Fig. 4(b) and Table 1. Next, we discuss the effects of initial gas production, gas production decline rate, and limited drilling year on optimization results using the control variable method.

**Table 1:** Parameters used in sensitivity analysis case.

Parameters	Value	Units
Production construction period	3	years
Stable production period	8	years
Production decline period	19	years
Target production	5 × 10^8^	m^3^/year
Drilling years	10	years
Initial gas production	10 × 10^4^	m^3^/d
Production decline rate	0.0005	1/d

### 3.1 Impact of initial gas production

Set the initial gas production of the gas well to 5 × 104m3/d, 8 × 104m3/d, 10 × 104m3/d, 12 × 104m3/d, 15 × 104m3/d, while maintaining the default parameters for the gas production decline rate and limited drilling year, which aims to examine the impact of the initial gas production on optimization results.

**Fig. 5:**
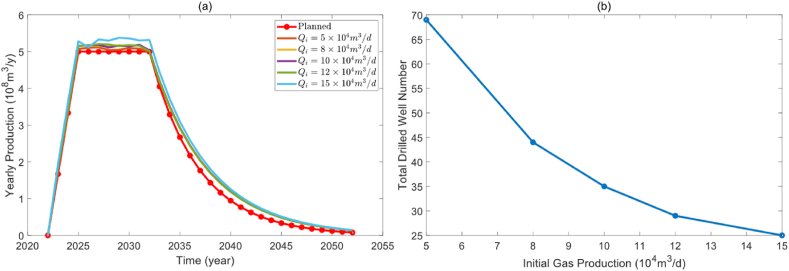
The effect of initial gas production. (a). Optimized production profile under different initial gas production. (b). Correlation between initial gas production and total drilled well number.

The impact of various initial gas production levels on the field production profile is illustrated in Fig. 5(a). It is clear that all results of the optimization model can well match the planned field production regardless of the baseline production. The extent of productivity overflow increases with the initial productivity of the gas well. However, from the perspective of the total number of drilled wells in Fig. 5(b), it gradually decreases with the increase of initial gas production. This is because, in the case of a constant decline rate, the greater the initial gas production, the greater the estimated ultimate recovery (EUR) of the gas well, and the fewer the total drilled wells required to meet the planned field production.

**Fig. 6:**
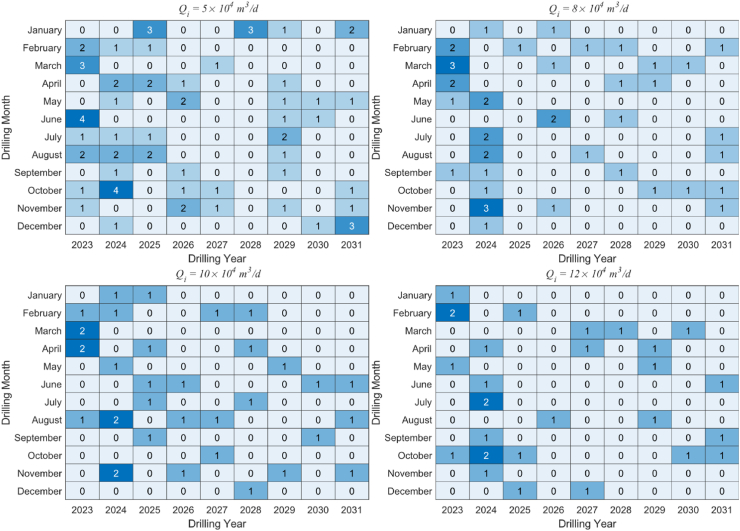
Heat map of drilling schedules under different initial gas production.

Drilling schedules are represented using heatmaps, which are statistical charts that depict data through colored blocks. Fig. 6 illustrates the effect of different initial gas production on the drilling schedule. Overall, due to the sparse processing of the drilling frequency and total number, the drilling frequency is not concentrated in a specific period of time but is scattered as much as possible at different times. However, what they have in common is that during the production construction period, the number of drilled wells in the first three years is relatively large to meet the planned field production as soon as possible. Once the stable production period is reached, new drilling will decrease, and the drilling density will also decrease.

### 3.2 Impact of production decline rate

**Fig. 7:**
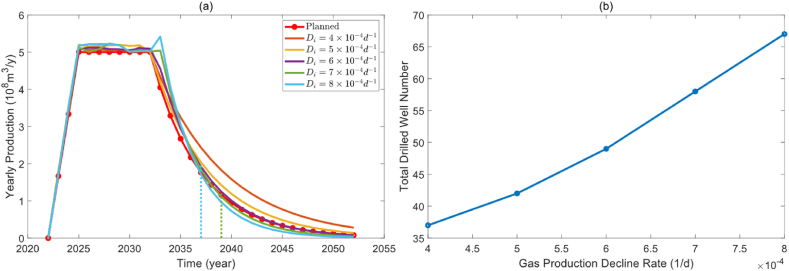
The effect of production decline rate. (a). Optimized production profile under different production decline rate. (b). Correlation between gas production decline rate and total drilled well number.

Another key parameter affecting the optimization results is the well decline rate, crucial for the production profile of both the well and the gas reservoir. Decline rates of 4 × 10-4/day, 5 × 10-4/day, 6 × 10-4/day, 7 × 10-4/day, and 8 × 10-4/day were set to investigate the impact of the production decline rate on the optimization results using default parameters for gas well production and limited drilling year. Fig. 7(a) illustrates the effects of production decline rate on the field production profile. With an increase in the production decline rate, the field production profile declines faster. If the decline rate is large in the later period of field production, it can no longer meet the planned field production. Fig. 7(b) illustrates how the production decline rate affects the total number of drilled wells. More drillings are necessary as the ultimate field production decreases when the production decline rate is high. Within a fixed limited drilling period, when the gas well production decline rate exceeds 7 × 10-4/d, the overall production profile can no longer meet the production demand during the decline period. During this time, even if drilling is increased during the stable production period, it will still be challenging to meet the production demand during the decline period.

**Fig. 8:**
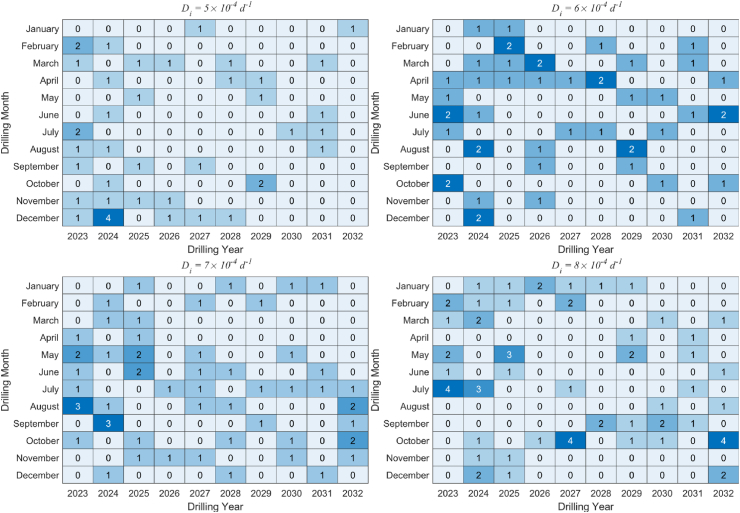
Heat map of drilling schedules under different production decline rate.

The heat map of drilling schedules for various production decline rates is depicted in Fig. 8. It is evident that as the production decline rate of gas wells increases, the color of the heat map gradually deepens, indicating an increase in drilling density. During the production construction period, production increases rapidly, resulting in more new drilled wells. New wells are more equally dispersed during the period of stable production, and they take the place of old wells to meet the production demands. Fewer new wells are needed to replace old wells for production when the production decline rate is small, and fewer wells are drilled towards the end of the limited drilling year.

### 3.3 Impact of limited drilling years

**Fig. 9:**
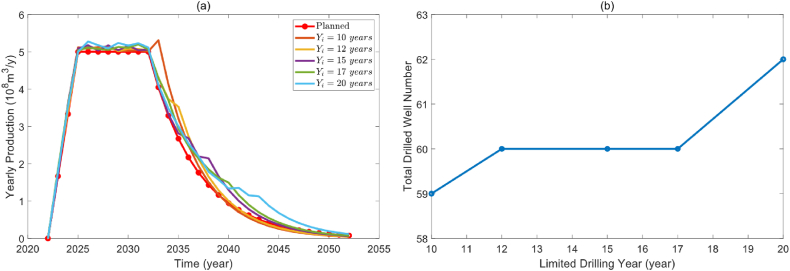
The effect of limited drilling year. (a). Optimized production profile under different drilling years. (b). Correlation between drilling years and total drilled well number.

Most gas reservoirs may not have long drilling years because longer drilling years require higher management expenditures. Fig. 9(a) shows the optimization results when the drilling years are 10 years, 12 years, 15 years, 17 years, and 20 years, respectively. Before the limited drilling year, all models can achieve the production target and meet the planned production demands. However, once the limited drilling year arrives, the field production begins declining to variable degrees because there is no new drilling to take over the production. Limited drilling years did not have a significant impact on the number of drilling wells. From the correlation between limited drilling years and total drilled number in Fig. 9(b), when the number of drillings is 60, and the limited drilling year increases from 12 to 17. The gas production profile of the optimized drilling sequence can meet the planned production profile, which also demonstrates the powerful optimization ability of the proposed model to meet the planned production profile with the minimum number of drilling wells.

**Fig. 10:**
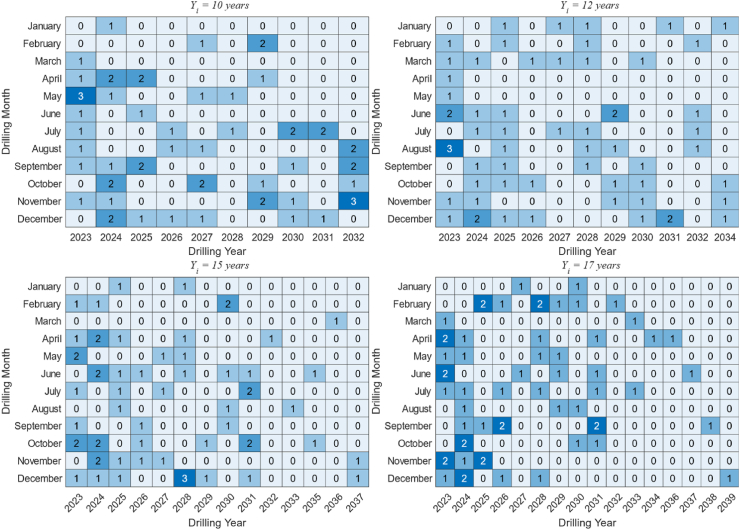
Heat map of drilling schedules under different limited drilling year.

The heat map of drilling schedules for various limited drilling years is displayed in Fig. 10. Drilling density decreases gradually as the limited drilling year increases, and the sequence of drillings gradually becomes uniform. Combined with the optimized field production profile, when the drilling period is short, the model will increase new drilled wells in the last year of the drilling period to meet the production demand in the decline period. The optimization model may systematically supplement drilling in the decline period to satisfy the field production demand when the drilling period is extended.

## 4 Field example

**Fig. 11:**
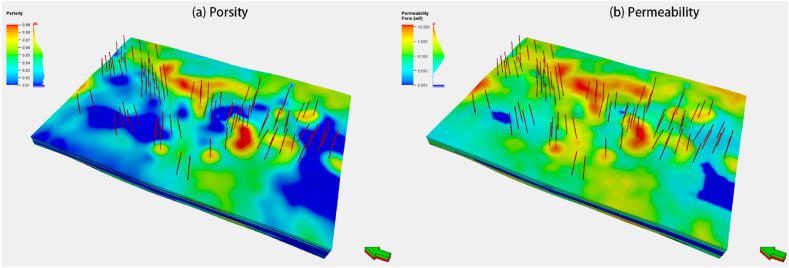
The geological model of the example gas reservoir. (a) Porosity model. (b) Permeability model.

While the preceding discussion is ideal, the production and decline of gas wells in a field often vary widely. Disparate gas well production curves pose a significant challenge to optimizing the model. In this example, the planned production profile of the gas reservoir is provided, while the production profile of gas wells is obtained through numerical simulation. The gas wells have completely different initial production and decline rates. The gas reservoir has a physical size of 3 km × 3 km × 40 m, and there are 97 potential drilled wells in the 30-year life cycle. Our goal is to select a reasonable number of drilled wells from the 97 potential drilled wells and design an optimal production sequence to achieve the planned field production profile. The porosity model and permeability model of the gas reservoir are shown in Fig. 11(a) and (b), respectively.

**Fig. 12:**
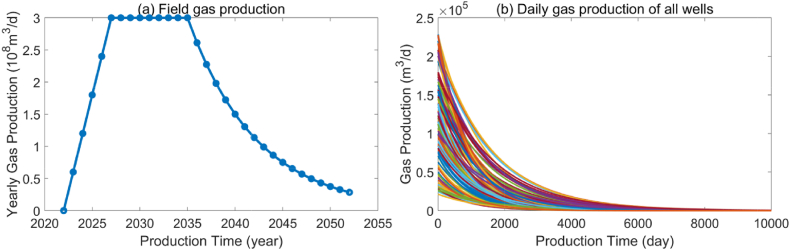
Planned production curves for gas reservoir (a) and production curves for all potential wells (b).

Fig. 12(a) depicts the planned field production profile. The period from 2023 to 2028 is the production construction period of gas reservoirs, from 2029 to 2035 is the stable production period of gas reservoirs, and from 2036 to 2052 is the decline period of gas reservoirs. The production profile of gas wells obtained by numerical simulation is displayed in Fig. 12(b). The initial production rate of the 97 gas wells is distributed between 1.2 × 104 m3/day and 24.3 × 104 m3/day, with an average of 12.5 × 104 m3/day. Each gas well has a unique decline rate.

**Fig. 13:**
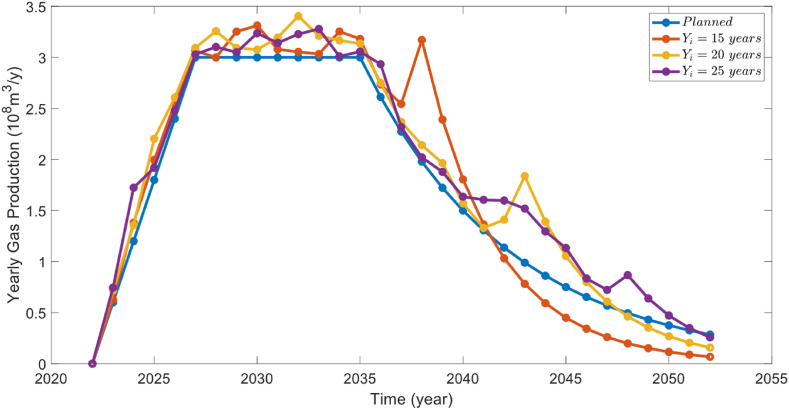
Field production profile under different limited drilling year.

We calculated the production profiles at 15 years, 20 years, and 25 years of drilling years, and the results are shown in Fig. 13. When the limited drilling years are 15 and 20, the production profile cannot be satisfied during the decline period because the production decline of the gas well is too fast in the late stage. As the drilling years increase, the optimization model gradually increases drillings during the decline period to compensate for the loss of production. With drilling years of 25 years, the optimized results met the planned field production requirements at all stages.

**Fig. 14:**
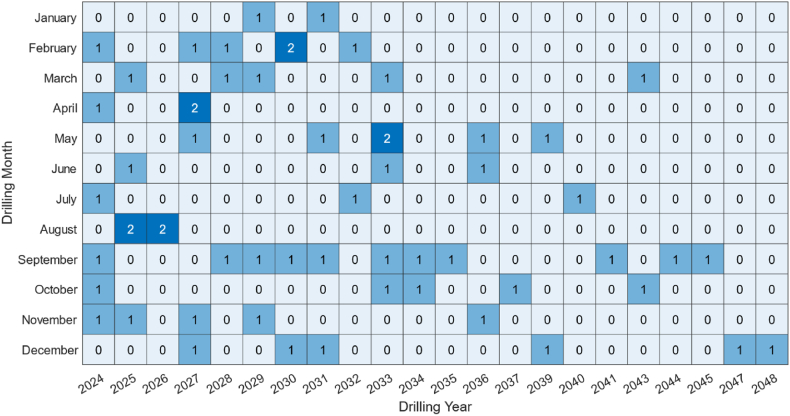
Drilling schedule heat map of field example.

Fig. 14 shows the heatmap of the drilling schedule with a limited drilling year of 25. It can be seen that the drilling schedule is aligned with the production profile, one by one. When the production profile is about to be lower than planned, new drillings will always be added to compensate for production and achieve the purpose of replacing old wells.

## 5 Conclusions

In this study, an optimization model for optimizing the summation of individual wells to meet the planned production is proposed. The field example yielded positive outcomes. Several conclusions can be drawn.

(1) The optimization model consumes memory space but achieves linearity, allowing it to be solved stably using the gradient descent algorithm.
(2) Key parameters, including initial gas production, production decline rate, and limited drilling years, are analyzed to understand their effects on the production profile and drilling schedule.
(3) A field example was conducted to demonstrate the effectiveness of the optimization model. With a drilling period of 25 years, the optimized results met the planned field production requirements at all stages.

## Data availability statement

All data, models, or code that support the findings of this study are available from the corresponding author upon reasonable request.

## CRediT authorship contribution statement

**Jingyun Ouyang:** Methodology, Software, Writing – original draft. **Shaoyang Geng:** Conceptualization, Writing – original draft, Writing – review & editing, Supervision. **Shuo Zhai:** Investigation, Visualization.

## Declaration of competing interest

The authors declare that they have no known competing financial interests or personal relationships that could have appeared to influence the work reported in this paper.

## Appendix A Solution to the Optimization Model

Here we show how to prove that the normalization will reduce the drilling frequency mathematically.

Obviously, L(D) in Eq. (12) is second-derivable. Let $D^{*}$ is the optimal solution of L(D), and 2nd order Taylor expansion of L(D) is given by:

(A.1) $$\hat{L}\left(D\right)=\hat{L}\left(D^{*}\right)+\frac{1}{2}{\left(D-D^{*}\right)}^{T}H\left(D-D^{*}\right)$$

In Eq. (A.1), **H** is the Hessian matrix of L(D) at $D^{*}$, which is (Eq. (A.2)):

(A.2) $$H=\left[\begin{array}{cc} \begin{array}{cc} \frac{\partial^{2}L\left(D^{*}\right)}{\partial^{2}D_{1,1,1}^{*}} & \frac{\partial^{2}L\left(D^{*}\right)}{\partial D_{1,1,1}^{*}\partial D_{1,2,1}^{*}}\\ \frac{\partial^{2}L\left(D^{*}\right)}{\partial D_{1,2,1}^{*}\partial D_{1,1,1}^{*}} & \frac{\partial^{2}L\left(D^{*}\right)}{\partial^{2}D_{1,2,1}^{*}} \end{array} & \begin{array}{cc} \cdots & \frac{\partial^{2}L\left(D^{*}\right)}{\partial D_{1,1,1}^{*}\partial D_{N,T_{dl},1}^{*}}\\ \cdots & \frac{\partial^{2}L\left(D^{*}\right)}{\partial D_{1,2,1}^{*}\partial D_{N,T_{dl},1}^{*}} \end{array}\\ \begin{array}{cc} \vdots & \vdots \\ \frac{\partial^{2}L\left(D^{*}\right)}{\partial D_{N,T_{dl},1}^{*}\partial D_{1,1,1}^{*}} & \frac{\partial^{2}L\left(D^{*}\right)}{\partial D_{N,T_{dl},1}^{*}\partial D_{1,2,1}^{*}} \end{array} & \begin{array}{cc} \ddots & \vdots \\ \cdots & \frac{\partial^{2}L\left(D^{*}\right)}{\partial^{2}D_{N,T_{dl},1}^{*}} \end{array} \end{array}\right]$$

Take the derivatives of Eq. (9) of each element in **D** and substitute them into Eq. (9), then we get Eq. (A.3):

(A.3) $$H=\left[\begin{array}{cc} \begin{array}{cc} \frac{\partial^{2}L\left(D^{*}\right)}{\partial^{2}D_{1,1,1}^{*}} & 0\\ 0 & \frac{\partial^{2}L\left(D^{*}\right)}{\partial^{2}D_{1,2,1}^{*}} \end{array} & \begin{array}{cc} \cdots & 0\\ \cdots & 0 \end{array}\\ \begin{array}{cc} \vdots & \vdots \\ 0 & 0 \end{array} & \begin{array}{cc} \ddots & \vdots \\ \cdots & \frac{\partial^{2}L\left(D^{*}\right)}{\partial^{2}D_{N,T_{dl},1}^{*}} \end{array} \end{array}\right]$$

Notice that:

(A.4) $$H=\frac{\partial^{2}L\left(D^{*}\right)}{\partial^{2}D_{N,k,1}^{*}}=2\sum_{y=1}^{Y_{pl}}\sum_{m=12y-11}^{12y}\sum_{i=1}^{N}\sum_{k=1}^{T_{pl}}G_{i,k,m}>0$$

According to Eqs. (A.3) and (A.4), **H** is a positive diagonal matrix.

Find the derivatives of Eq. (A.1) with respect to **D**, which is given by:

(A.5) $$\nabla_{D}\hat{L}\left(D\right)=H\left(D-D^{*}\right)$$

Similarly, find the derivatives of Eq. (12) of **D**:

(A.6) $$\nabla_{D}\hat{J}\left(D\right)=\nabla_{D}\hat{L}\left(D\right)+\alpha \text{sign}\left(D\right)$$

where sign(∙) is the sign function. When x<0, sign(x)=−1; When x=0, sign(x)=0; When x>0, sign(x)=1.

Let Eq. (A.6) equals to 0, with Eq. (A.5), and the optimal solution $\tilde{D}$ of the final object function satisfies (Eq. (A.7)):

(A.7) $$H\left(\tilde{D}-D^{*}\right)+\alpha \text{sign}\left(\tilde{D}\right)=0$$

With Eq. (34), each element of $\tilde{D}$ is given by Eq. (A.8):

(A.8) $${\tilde{D}}_{i,k,1}=\text{sign}\left(D_{i,k,1}^{*}\right)\max\left(\left|D_{i,k,1}^{*}\right|-\frac{\alpha }{H},0\right)$$

Combine Eqs. (A.4)(A.8), and the solution is shown in Eq. (A.9):

(A.9) $${\tilde{D}}_{i,k,1}=\left\{ \begin{array}{c} 0,\left|D_{i,k,1}^{*}\right|\leq \frac{\alpha }{H}\\ sign\left(D_{i,k,1}^{*}\right)\left(\left|D_{i,k,1}^{*}\right|-\frac{\alpha }{H}\right),\left|D_{i,k,1}^{*}\right|>\frac{\alpha }{H} \end{array}\right.$$
